# Unmasking Neurosarcoidosis in a Patient With Acute Meningitis Features

**DOI:** 10.7759/cureus.97633

**Published:** 2025-11-24

**Authors:** Tun Tun, Tharaka Premarathne, Iman Al-Rifaie

**Affiliations:** 1 Acute General Medicine, Horton Hospital, Oxford University Hospitals NHS Foundation Trust, Banbury, GBR

**Keywords:** diagnostic challenge, immunosuppression, meningitis, meningoencephalitis, neurosarcoidosis

## Abstract

Neurosarcoidosis is a rare disorder that may mimic infectious, inflammatory, and neoplastic conditions, therefore making diagnosis challenging. We report the case of a 32-year-old man with a background history of Crohn’s disease treated with Adalimumab who presented with a 10-day history of worsening headache, confusion, and new-onset seizures. He also experienced transient sensory changes with one episode of fever. Neurological examination was unremarkable. Given his immunosuppressed condition, empirical treatment for meningoencephalitis was initiated. Initial CT scan of the brain was normal, while the first lumbar puncture demonstrated mild pleocytosis (18 × 10^6^/L, predominantly lymphocytic) but negative cultures. MRI scan of the brain revealed multifocal white matter lesions, initially suggestive of demyelination. A second lumbar puncture showed a significant rise in white cell count (110 × 10^6^/L, predominantly lymphocytic), but infectious and autoimmune screens remained negative. Given a persistent headache despite broad-spectrum antimicrobials, further workup was pursued. CT venography excluded venous sinus thrombosis. Surprisingly, systemic imaging revealed bilateral hilar and mediastinal lymphadenopathy. Endobronchial ultrasound-guided biopsy confirmed non-caseating granulomatous inflammation consistent with sarcoidosis, with no evidence of lymphoma or tuberculosis. We requested CSF angiotensin-converting enzyme levels retrospectively, and the result was elevated, further supporting the diagnosis of neurosarcoidosis. After multidisciplinary team review, high-dose intravenous methylprednisolone was commenced, followed by an oral prednisolone taper, leading to significant symptomatic improvement.

This case highlights the complex diagnostic pathway of neurosarcoidosis, particularly in immunosuppressed patients, where infection is the predominant concern. The overlapping clinical and radiological features with meningoencephalitis and demyelinating disease emphasise the need to consider neurosarcoidosis in the differential diagnosis. Early multidisciplinary involvement, systematic exclusion of infection and malignancy, and timely initiation of immunosuppression were critical to achieving a favourable outcome in this case. Clinicians should maintain a high suspicion for neurosarcoidosis in patients presenting with atypical neurological features, as early recognition and treatment may substantially alter prognosis.

## Introduction

Sarcoidosis is a multisystem granulomatous disorder, characterised histologically by non-caseating granulomas. The aetiology of sarcoidosis is unknown. Lungs are mostly affected in nearly 90% of patients. Other commonly affected sites include the skin (up to 30%), eyes (10-25%), and mediastinal or hilar lymph nodes (90%) [1]. Approximately 5-10% of patients have neurological involvement, referred to as neurosarcoidosis, and may present with meningitis, cranial neuropathies, seizures, or extremity weakness [2].

In neurosarcoidosis, the most frequently affected areas are cranial nerves, especially the facial nerve; however, it can also present with polyneuropathy, hydrocephalus, meningitis, myelopathy, or hypothalamic-pituitary dysfunction. In 62-74% of cases, neurological symptoms are the initial clinical manifestation of sarcoidosis, followed by systemic features of sarcoidosis [3]. Less than 1% of cases are presented with isolated neurosarcoidosis without features of systemic sarcoidosis [2].

Although neurosarcoidosis has an association with environmental exposures (infectious agents, inorganic compounds, agricultural factors) and genetic factors (e.g., annexin A11 and BTNL2 mutations), the exact pathophysiology remains unclear [4].

Getting the diagnosis of neurosarcoidosis is challenging due to the diversity of neurological presentations and the need to exclude other granulomatous conditions such as lymphoproliferative disease, infections, drug reactions, and autoimmune conditions [5]. In addition, biopsy of the nervous system is often impractical, requiring indirect evidence for diagnosis. A combination of neuroimaging, CSF evidence of central nervous system inflammation, histological confirmation of systemic sarcoidosis, or indirect markers such as FDG-PET, gallium scan, or elevated serum angiotensin-converting enzyme (ACE) supports a diagnosis of neurosarcoidosis [6].

Therefore, the diagnosis of sarcoidosis depends on three major criteria: compatible clinical presentation, demonstration of non-necrotising granulomatous inflammation, and the exclusion of alternative causes [7].

Here, we report the case of a young man on immunosuppressant condition who presented with neurological symptoms such as seizures, progressive headache, and confusion, and was ultimately diagnosed with neurosarcoidosis.

## Case presentation

A 32-year-old man with a background of Crohn’s disease treated with Adalimumab presented to the emergency department with a 10-day history of worsening throbbing headache, progressive confusion, and one episode of generalised seizure. His parents also reported that he was unable to perform routine tasks he previously knew. He had transient sensory changes affecting the right arm, face, and teeth for three days, along with a single episode of fever before admission. On arrival, neurological examination was unremarkable.

**Figure 1:**
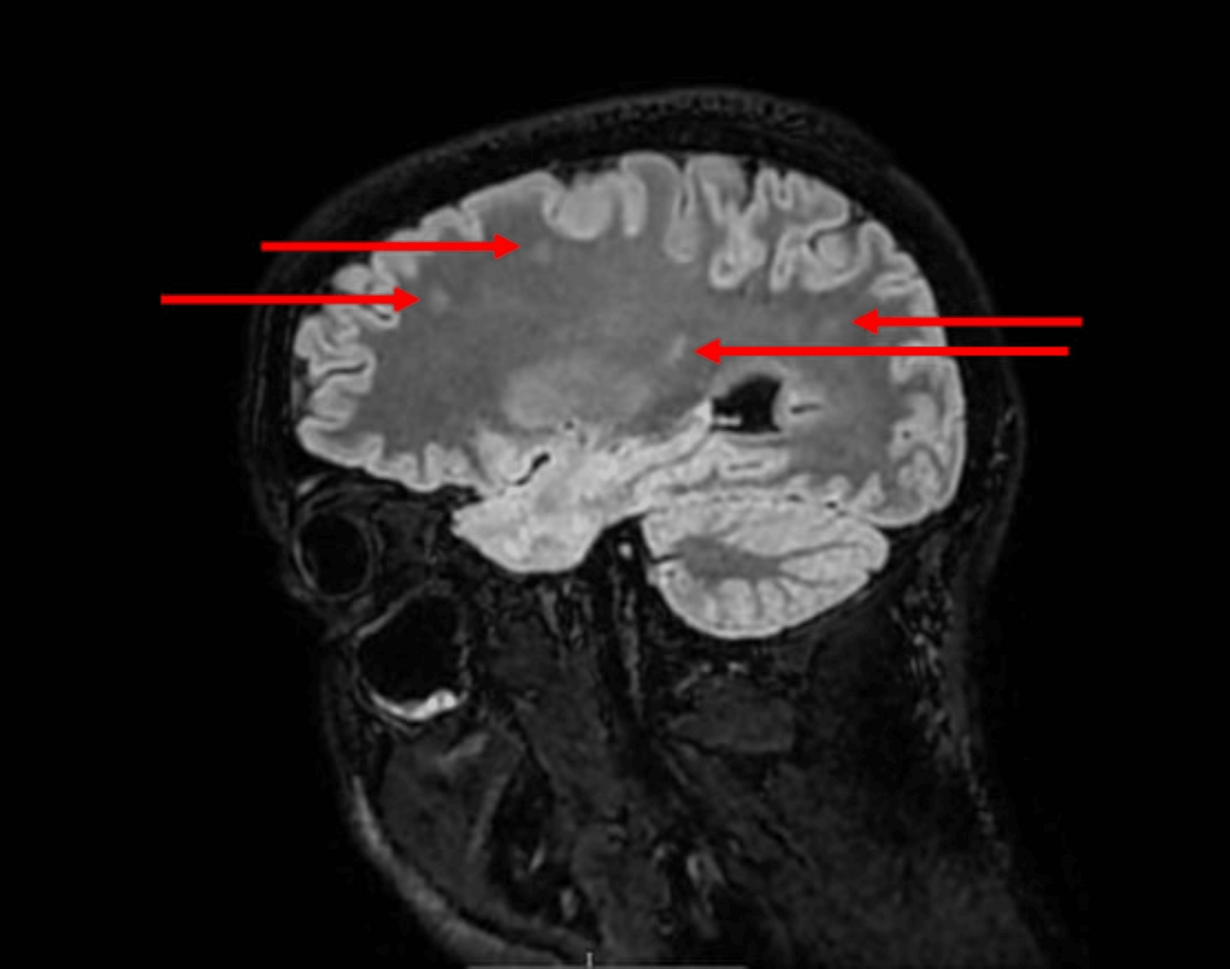
Brain MRI Brain MRI showing multifocal white matter lesions (red arrows).

Given his immunosuppressed status, empirical intravenous amoxicillin, ceftriaxone, and acyclovir were commenced for suspected meningoencephalitis. Initial CT brain showed no acute pathology. The first lumbar puncture revealed mild pleocytosis (18 × 10^6^/L, eight polymorphs and 10 lymphocytes), with negative bacterial and mycobacterial cultures (Table 1). Despite treatment, his headache persisted. MRI brain demonstrated multifocal white matter lesions in the right inferior frontal and left temporal juxtacortical regions, the right hemi-pons, and posterior medulla, initially thought to represent demyelinating plaques (Figure 1).

**Table 1:** Blood tests excluding infections and autoimmune conditions ACE, angiotensin converting enzyme; ALT, alanine aminotransferase; AMPA antibody, α-amino-3-hydroxy-5-methyl-4-isoxazolepropionic acid antibody; APTT, activated partial thromboplastin time; CASPR2 antibody, contactin-associated protein-like 2 antibody; CMV, cytomegalovirus; CRP, C-reactive protein; CSF, cerebrospinal fluid; EBV, Epstein-Barr virus; FBC, full blood count; GABA(a)R antibody, gamma-aminobutyric acid type A receptor antibody; GABA(b)R antibody, gamma-aminobutyric acid type B receptor antibody; HBsAg, hepatitis B surface antigen; HCV Ab, hepatitis C virus antibody; HEV IgG/IgM, hepatitis E virus immunoglobulin G and immunoglobulin M; HIV, human immunodeficiency virus; IgG, immunoglobulin G; IgM, immunoglobulin M; IGRA, interferon-gamma release assay; INR, international normalized ratio; IU/L, international unit per litre; JC, John Cunningham virus; LGI1 antibody, leucine-rich glioma-inactivated 1 antibody; mg/L, milligram per litre; mmol/L, millimole per litre; NMDA receptor Ab, N-methyl-D-aspartate receptor antibody; PCR, polymerase chain reaction; RNA, ribonucleic acid; RSV, respiratory syncytial virus; µmol/L, micromole per litre; µmol/min/L, micromole per minute per litre; VHF, viral haemorrhagic fever Table created by the authors.

Parameters		Readings	Normal Ranges
FBC	Haemoglobin	156	130-170 g/L
	White cell count	8.98	4-11 × 10^9^/L
	Platelet	205	150-400 × 10^9^/L
Clotting screening	Prothrombin time	11.6 seconds	9-12 seconds
	APTT	22.6 seconds	20-30 seconds
	INR	1.1	-
Electrolytes	Sodium	137 mmol/L	133-146 mmol/L
	Potassium	4 mmol/L	3.5-5.3 mmol/L
	Urea	6.3 mmol/L	2.5-7.8 mmol/L
	Creatinine	79 µmol/L	64-104 µmol/L
Liver function test	Total bilirubin	12 µmol/L	0-21 µmol/L
	ALT	33 IU/L	10-35 IU/L
	Alkaline phosphatase	59 IU/L	30-130 IU/L
	CRP	2 mg/L	0-5 mg/L
First CSF result	CSF white cell count	18 × 10^6^/L	-
	CSF polymorphs	8 × 10^6^/L	-
	CSF lymphocytes	10 × 10^6^/L	-
	CSF protein	859 mg/L	0-400 mg/L
	CSF glucose	3.5 mmol/L	-
	CSF lactate	1.9 mmol/L	1.1-2.4 mmol/L
	CSF bacterial culture	Negative	-
	CSF mycobacterial culture	Negative	-
	CSF viral PCR	Negative	-
	CSF cryptococcal antigen	Negative	-
	CSF ACE level (retrospectively requested)	2.06 µmol/min/L	0-1.2 µmol/min/L
Second CSF result	CSF white cell count	110 × 10^6^/L	-
	CSF polymorphs	20 × 10^6^/L	-
	CSF lymphocytes	90 × 10^6^/L	-
	CSF protein	1064 mg/L	0-400 mg/L
	CSF bacterial culture	Negative	-
	CSF glucose	2.7	-
	CSF lactate	1.8 mmol/L	1.1-2.4 mmol/L
	CSF oligoclonal band	Present	-
	CSF mycobacterial c	Negative	-
	CSF JC virus PCR	Negative	-
	CSF viral PCR	Negative	-
Third CSF result	CSF white cell count	100 × 10^6^/L	-
	CSF polymorphs	18 × 10^6^/L	-
	CSF lymphocytes	82 × 10^6^/L	-
Infection screening	HIV antibody	Negative	-
	Syphilis serology	Negative	-
	Borrelia burgdorferi IgG	Negative	-
	Cryptococcal antigen	Negative	-
	Blood culture	Negative	-
	Legionella urine antigen	Negative	-
	Respiratory viral PCR	Negative	-
	COVID-19 PCR	Negative	-
	Influenza virus PCR	Negative	-
	RSV PCR	Negative	-
	CMV IgG/IgM	Negative	-
	EBV IgG	Negative	-
	HBsAg	Negative	-
	HCV Ab	Negative	-
	HEV IgG/IgM	Negative	-
	VHF screening	Negative	-
	Rickettsia Ab	Negative	-
	Zika virus	Negative	-
	Chikungunya IgG/IgM/RNA	Negative	-
	Dengue IgG/IgM/RNA	Negative	-
	Flavivirus IgG	Negative	-
	West Nile virus IgM	Negative	-
	TB Elispot IGRA test	Negative	-
	Enterovirus screening, faeces	Negative	-
	Beta-D-glucan Ag level	Negative	-
Transbronchial needle aspirate	Bacterial culture	Negative	-
	Fungal culture	Negative	-
	Microscopy	Non-necrotising granulomatous inflammation	-
	Mycobacterium culture	Negative	-
Autoimmune screening	AMPA 1/2 receptor antibody CSF	Negative	-
	CASPR2 antibody CSF	Negative	-
	GABA (b) R antibody CSF	Negative	-
	LGI1 antibody CSF	Negative	-
	Fixed cell NMDA receptor Ab CSF	Negative	-
	GABA (a) R antibody CSF	Negative	-
	Myelin oligodendrocyte (MOG) Ab serum	Negative	-
	Aquaporin-4 Ab level, blood	Negative	-
	Anti-nuclear Ab level, blood	Negative	-
	Gastric parietal cell Ab level, blood	Positive	-
	Smooth muscle IgG Ab level	Negative	-
	Liver and kidney microsomal Ab	Negative	-
	Mitochondrial Ab level, blood	Negative	-
	Intrinsic factor Ab level, blood	Negative	-

A repeat lumbar puncture performed to evaluate for autoimmune encephalitis showed marked lymphocytic pleocytosis (110 × 10^6^/L, 90 lymphocytes) but negative antibody and microbiological panels (Table 1).

On day 7, due to ongoing symptoms, CT venography was performed to exclude venous sinus thrombosis, given his underlying Crohn’s disease, and this was negative. As part of an extended infectious disease evaluation, doxycycline was added to cover Rickettsial infection owing to recent travel to Tenerife. However, full blood count and CRP remained within normal range, and infectious screens remained negative.

**Figure 2:**
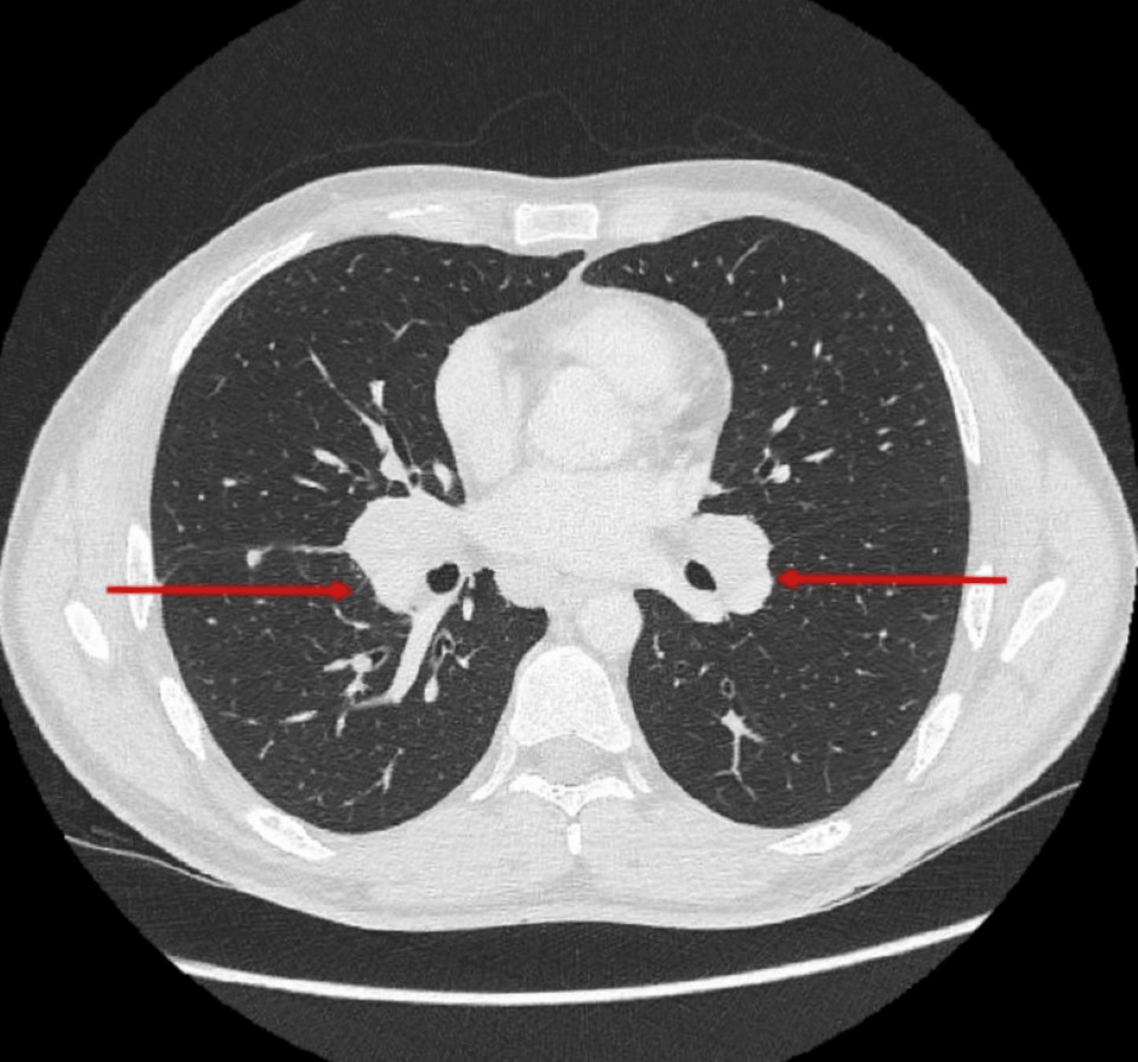
Chest CT Chest CT showing bilateral hilar lymphadenopathy (red arrows).

The case was discussed at a multidisciplinary meeting, where the possibility of CNS lymphoma was raised in view of MRI white matter lesions. Systemic imaging was therefore performed. CT chest, abdomen, and pelvis unexpectedly revealed bilateral hilar and mediastinal lymphadenopathy (Figure 2).

Endobronchial ultrasound-guided biopsy confirmed non-caseating granulomatous inflammation consistent with sarcoidosis, with no evidence of lymphoma or tuberculosis (Table 1). Retrospective testing showed elevated CSF ACE, further supporting the diagnosis of neurosarcoidosis (Table 1).

After a second MDT discussion involving neurology, neuroradiology, respiratory, and infectious disease specialists, high-dose intravenous methylprednisolone 500 mg once a day for five days was initiated. The patient demonstrated progressive improvement in headache and cognitive function, and no additional seizure episodes occurred following the first dose of methylprednisolone. He completed a five-day course of intravenous methylprednisolone followed by a tapering course of oral prednisolone. At follow-up, he remained clinically stable with resolution of neurological symptoms, and long-term immunosuppression is being considered to maintain remission. At the time of writing this case report, he remains on a tapering dose of oral prednisolone.

This case illustrates the complexity of diagnosing neurosarcoidosis, particularly in an immunosuppressed patient, where infection and malignancy were initially prioritised. The overlapping clinical and radiological features delayed diagnosis until systemic sarcoidosis was demonstrated. Early multidisciplinary involvement and timely initiation of corticosteroids were critical in achieving a favourable outcome.

## Discussion

Sarcoidosis is a systemic inflammatory disease characterised by noncaseating granulomas, affecting multiple organ systems. It occurs mostly among women and individuals of African descent in North America; however, isolated neurosarcoidosis without systemic involvement remains extremely rare, occurring in less than 1% of patients [1]. In neurosarcoidosis, a hallmark feature is cranial neuropathy; however, our patient presented with recurrent acute headaches, central fever, and pachymeningitis, which are atypical manifestations, making the diagnosis a challenge [2].

In neurosarcoidosis, both the central and peripheral nervous systems are affected, presenting with cranial neuropathies, aseptic meningitis, seizures, neuropsychiatric symptoms, neuroendocrine dysfunction, and peripheral neuropathies [5,8]. Leptomeningeal involvement typically involves the skull base, hypothalamus, and pituitary gland, while in spinal sarcoidosis, the neuroimaging features mimic intramedullary tumours on imaging [9]. In our patient, neuroimaging demonstrated diffuse dural thickening consistent with pachymeningitis, without cranial nerve involvement, making broader differentiation from infectious or neoplastic etiologies essential. Furthermore, other differential diagnoses such as tuberculosis, fungal infections, lymphoma, and autoimmune disorders need to be excluded [9,10].

The pathogenesis of neurosarcoidosis is not completely understood but likely secondary to dysregulated immune responses in genetically predisposed individuals, with triggering from environmental factors such as infections (mycobacteria and viruses), neoplasms, occupational exposures (e.g., agricultural employment or insecticides used at work), and inorganic compounds (e.g., aluminium) [4]. The mechanisms underlying neurological involvement in neurosarcoidosis are not fully elucidated, although inflammatory cytokine upregulation, oxidative stress, and alterations in neurotransmitter pathways have been proposed to contribute to neurological deficits [5]. Genetic mutations in ANXA11 and BTNL2 appear to influence susceptibility and disease progression in sarcoidosis. Moreover, certain genetic variants have been associated with specific clinical phenotypes, such as HLA-DRB10301 and TGF-β3 polymorphisms predisposing to Löfgren’s syndrome and pulmonary fibrosis, respectively. Certain nervous system involvement in sarcoidosis may also reflect genetic predisposition. For example, Caucasians are more susceptible to developing peripheral nervous system manifestations [5].

Histologically, the non-caseating granuloma is the hallmark of sarcoidosis. Sarcoid granulomas are composed of macrophages, epithelioid cells, multinucleated giant cells, and lymphocytes, with peripheral lymphopenia often observed due to T cell sequestration [5]. Therefore, tissue biopsy is needed for establishing a definitive diagnosis of sarcoidosis. However, in neurosarcoidosis, it is often difficult to access CNS tissue biopsy, so an extraneural biopsy, such as mediastinal lymph node sampling performed in our patient, can be used for diagnosis [9, 11]. Serum and CSF ACE levels may be elevated and can be used as supporting evidence for sarcoidosis, but their sensitivity is limited, though in our patient, significant CSF ACE elevation supported the diagnosis after infectious and malignant causes were excluded [3,12]. 

Management of neurosarcoidosis remains challenging due to the lack of randomised controlled trials. Corticosteroids are the mainstay of therapy, often requiring high doses for disease control. Other immunosuppressive agents, such as methotrexate, azathioprine, cyclophosphamide, or biologics, are reserved for refractory cases [13]. Early recognition after exclusion of infectious causes and other autoimmune causes, and initiation of treatment, are crucial to prevent irreversible neurological damage. In our patient, after initiation of corticosteroid, his neurological symptoms responded to corticosteroid favourably, and there was a gradual symptom improvement.

This case represents the rarity of isolated neurosarcoidosis, particularly presenting with pachymeningitis, recurrent headaches, and fever without cranial neuropathy, and also highlights the importance of maintaining a high index of suspicion in patients with atypical neurological presentations. Careful exclusion of infectious, neoplastic, and autoimmune mimics, combined with histopathological confirmation, supportive CSF and imaging findings, and multidisciplinary team involvement, facilitated the diagnosis and guided effective therapy [6,7,14].

## Conclusions

Neurosarcoidosis can present with atypical presentation, and it remains a diagnostic challenge due to its diverse neurological manifestations and its ability to mimic infectious, inflammatory, and neoplastic disorders. This case highlights the importance of maintaining a high index of suspicion, particularly in immunosuppressed patients presenting with atypical neurological symptoms. A systematic exclusion of infection, autoimmune condition and malignancy, recognition of supporting systemic features, and multidisciplinary collaboration are essential for timely diagnosis. Early initiation of corticosteroid therapy led to a favourable clinical outcome in our patient, highlighting the value of prompt immunosuppressive treatment once neurosarcoidosis is established. Ongoing follow-up, monitoring the features of other systemic sarcoidosis and consideration of long-term steroid-sparing immunomodulatory therapy are important to prevent relapse and disease progression.
